# Polymer-based ultrawideband transducers for high resolution hemispherical optoacoustic tomography

**DOI:** 10.1038/s41377-025-02101-8

**Published:** 2026-01-01

**Authors:** Amanda P. Siegel, Rayyan Manwar, Kamran Avanaki

**Affiliations:** 1 Richard and Loan Hill Department of Biomedical Engineering, University of Illinois at Chicago, Chicago, IL USA; 2 Section of Neonatology, Department of Pediatrics, UI Health Children’s Hospital of the University of Illinois at Chicago, Chicago, IL USA; 3 Department of Dermatology, University of Illinois at Chicago, Chicago, IL USA

**Keywords:** Photoacoustics, Biophotonics

## Abstract

Available transducers do not fulfill all of the necessary design criteria for high-performance hemispherical optoacoustic tomography, namely: an ultrawide bandwidth in order to acquire the full range of optoacoustic emissions from targets of interest, good impedance matching to minimize reverberation artifacts, and a modifiable form factor, for inclusion in non-flat geometries. Polyvinylidene fluoride (PVDF) transducers can, in principle, meet all of these criteria, but PVDF has known shortcomings. In *Ultrawideband high-density polymer-based spherical array for functional optoacoustic micro-angiography*, all of the challenges of working with PVDF are overcome with the demonstration of a high-performance PVDF-based hemispherical optoacoustic tomographic system.

## Abstract (web summary)

A representation of ultrawideband PVDF/co-polymer-based ultrasound (US) transducer technology for high-frequency hemispherical optoacoustic tomography and its potential use in hand vascular, dental, ophthalmic, and endoscopic applications.

## Graphical abstract

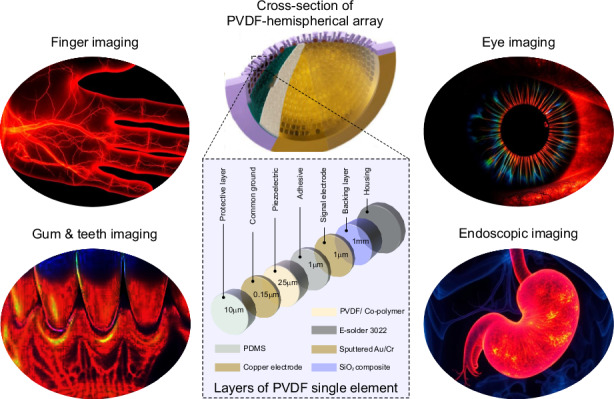

There is a growing recognition that one major roadblock to improving optoacoustic (OA) imaging performance is the limited capabilities of existing transducers. These transducers were most often designed for ultrasound (US) imaging, which has a different set of requirements than OA imaging, where transducers operate only in receiver mode. First, US transducers must have high electromechanical coupling efficiency to be able to generate strong, consistent acoustic signals. However, because OA imaging does not rely on transducer-generated acoustic waves, this property is not relevant for OA imaging. Second, in US imaging, the signal received will have a very similar bandwidth as the signal generated, so there is no strong need for wideband transducers. However, because OA signals are generated by the thermoelastic effect, there is no limitation on how wide a frequency range they can encompass. Third, in US imaging, although reverberations should be minimized as they reduce overall signal strength, because the transmitted signal is known, it is possible to utilize developed, effective methods for reducing the impact of reverberations (active control, pulse sequencing, time gating, and established correction methods). However, because OA imaging generates weaker signals and cannot use transmission-matching techniques for reducing reverberation effects, it has a greater need to reduce or eliminate reverberations (i.e. improve transducer/tissue impedance matching). OA imaging is typically limited to using ultrasound (US) transducers, not because they are ideal, but because they are the most readily available option. But what if an OA tomographic system were designed with transducers optimized for OA imaging rather than US imaging? How much would this impact OA image reconstruction?

The core materials for ultrasound transducers are either piezo-ceramics or piezo-polymers. Polyvinylidene fluoride (PVDF) is a ferroelectric polymer that was first recognized to have strong piezoelectric properties over 50 years ago^1^. PVDF-based transducers can detect a wide range of frequencies (up to 70 MHz or more) with an extremely wide bandwidth (>100%). Their mechanical flexibility allows them to be fabricated in various shapes and curvatures. Their impedance (around ~4 MRayl) is much closer to tissue (~1.5 MRayl) than typical piezoceramic transducers (lead zirconate titanate (PZT) has impedance of ~33 MRayl), which gives PVDF transducers improved reception sensitivity and leads to fewer reverberation-induced artifacts^2^. Despite these features, PVDF transducers do have a number of shortcomings, including the material’s relative fragility (both mechanical and thermal), a low capacitance requiring carefully designed front-end electronics, and the need for preamps. These, as well as its lower electromechanical coupling efficiency compared with PZT^3^, have discouraged the development of PVDF transducers for US imaging.

About 8 years ago, Razansky’s group fabricated a single-shot hemispherical OA microtomography setup based on PZT detectors for use as an ultrafast multi-scale functional OA tomography (OAT) system^4^. 510 PZT sensing elements were fabricated on seven planar segments in a “flower” geometry (one central segment surrounded by six petals, which are bent into an approximate hemispherical surface). Isotropic spatial resolution of around 35 μm was achieved using the system with a detection bandwidth of ~10-30 MHz (high frequency). It is known that small structures generate strong OA high-frequency signals, while large structures generate weak high-frequency signals, attributable to embedded small features within the large structures. An OA system that includes low frequencies can generate strong OA signals from the large structures. For this reason, in the discussion of this article, they noted that improving the performance of their system “would necessitate the development of ultrasonic arrays with larger detection bandwidth”^4^. The Razansky group then developed a collaboration with Subochev, an expert at the fabrication of PVDF-based detectors^5^, to design OA imaging systems with ultrawideband transducers^6,7^.

In a newly published paper in Light: Science & Applications, Razansky and Subochev describe the latest results of their collaboration^8^. The new imaging system delivers real-time multi-scale volumetric imaging with 35 μm lateral/22 µm axial resolution and high image fidelity. The paper shows how this system is able to perform spectroscopic, time-resolved, volumetric imaging in live mice to identify stimulation-based regiospecific cerebral oxygenation changes within a 0.3 × 0.3 × 0.1 mm^3^ volume of interest” and also demonstrates high resolution volumetric image of human palm vasculature with vessel diameters ranging from 50 to 500 μm. To further illustrate the advantages of their system, the authors compared their current system with Razansky’s previous PZT system (described in Deán-Ben et al.^4^). For a 1.5 mm agar phantom, the PVDF system delivered much better sensitivity to low (0.3–5 MHz, ~24 dB SNR increase) and medium (5–25 MHz, ~11 dB SNR increase) frequencies compared with the PZT equivalent^8^.

Fabrication of the PVDF spherical array, incorporation of the pre-amplification electronics, and array calibration required meticulous attention to detail. For example, fabrication started with a hemispherical preform with 512 cavities and 512 copper electrodes, both manufactured with very high precision. After soldering individual micro-coaxial wires to each electrode, the electrodes were mounted onto the 512 cavities of the preform and fixed in place with a custom-made epoxy-based compound with low impedance (matching PVDF) to avoid reverberations. Next, a 25 μm thick sheet of PVDF was stretched around a steel ball to attain the hemispherical shape, and glued to the inner surface of the preform, and a metallic electrode film of chrome and gold was sputter-coated on top of the concave detection system to form the ground electrode. To boost the signal, each of the 512-element electrodes required a matching amplification channel (two-stage amplification circuit and emitter follower), which was shielded and located as close as possible to the individual array element. Power consumption for the amplifier led to the need to build a forced air ventilation system with a forced amplifier shutdown for overheating (this is a very relevant concern, because PVDF begins to melt around 80 °C). The outcome of this significant feat of engineering, however, was high spatial resolution and high image fidelity achieved over a wide range of target sizes.

In addition to the setup described in Subochev et al.^8^, PVDF films could also be ideal for applications that require a small form factor and diverse shape and curvature, such as finger imaging (for biometric identifications or for assessing synovitis^9,10^), ophthalmic imaging (for angiography, sO_2_ and pigment imaging^11,12^), dental imaging (for improved detection of caries, gum disease, and apical healing^13,14^), endoscopic imaging (for gastrointestinal tract and the cervical canal monitoring^15,16^), and for applications involving molecular tracking of exogenous contrast agents and spectroscopic tissue analysis^17^. Potential uses of PVDF/copolymer-based transducers and its typical component layers are shown in Fig. 1. An alternate application involves stacking a PVDF transducer onto a PZT transducer to enable transmission (by PZT) and reception (by PVDF) of short-pulsed ultrasound from the same location (e.g., for monitoring ultrasound microbubbles)^18^. Stacked systems could replace dual US/OA imaging setups currently designed with interleaved low-frequency and high-frequency transducers (to achieve ultrawideband resolution), such as described in Zhao et al.^19^. However, the stacking mechanism requires multiple matching layers for relative transitioning from high (PZT) to low impedance (PVDF), which makes the transducer bulkier and less suitable for hemispherical designs. Alternatively, US transmission capability can be achieved via enhancing piezoelectric properties of PVDF by blending with polymers or adding fillers while maintaining a small form factor and reduced fabrication complexity^20^. Poly(vinylidene fluoride-trifluoroethylene) (PVDF-TrFE), a commercially available copolymer of PVDF, has a better electromechanical coefficient, higher thermal stability, and very low mechanical and electrical loss, but perhaps cannot achieve the same ultrawideband sensitivity in comparison to conventional PVDF^21–23^. Looking forward, the main impact of *Ultrawideband high density polymer-based spherical array for functional optoacoustic micro-angiography* is that it demonstrates that researchers in OA imaging should not have to accept the limitations imposed by the use of transducers designed for US imaging. It also highlights the urgent need for the field to develop polymer-based transducers specifically for OA imaging.

**Fig. 1:**
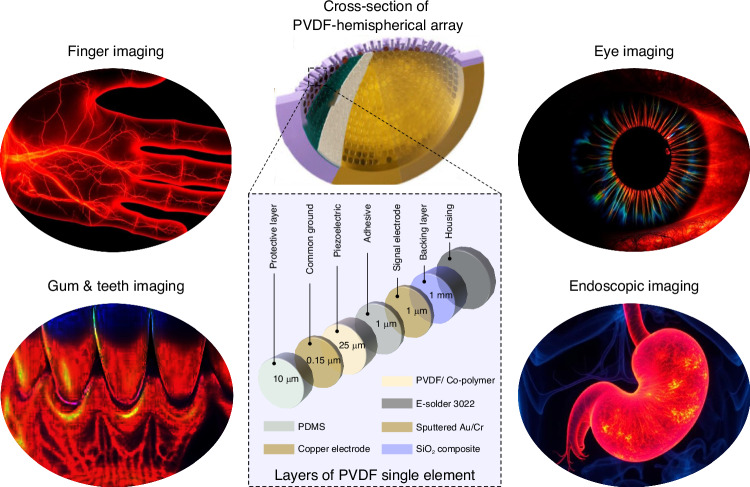
A representation of ultrawideband PVDF/ co-polymer-based ultrasound (US) transducer technology for high frequency hemispherical optoacoustic tomography and its potential use in hand vascular, dental, ophthalmic, and endoscopic applications
